# AMIGO2 as a Novel Biomarker Predicting Poor Prognosis and Associated with Adhesion-Driven Metastasis in Pancreatic Adenocarcinoma

**DOI:** 10.7150/ijms.121794

**Published:** 2026-01-14

**Authors:** Ze-Syuan Chen, Tsung-Shun Chang, Wan-Jou Shen, Shan-Ju Liu, Chih-Yang Wang, Yung-Kuo Lee, Wen-Hsin Hsu, Wei-Jan Wang

**Affiliations:** 1 Department of Biological Science and Technology, College of Life Sciences, China Medical University, Taichung, Taiwan ROC.; 2 Department of Otolaryngology, Kaohsiung Armed Forces General Hospital, Kaohsiung, Taiwan ROC.; 3 Graduate Institute of Biomedical Sciences, China Medical University, Taichung, Taiwan ROC.; 4 Graduate Institute of Cancer Biology and Drug Discovery, College of Medical Science and Technology, Taipei Medical University, Taipei, Taiwan ROC.; 5 Ph.D. Program for Cancer Molecular Biology and Drug Discovery, College of Medical Science and Technology, Taipei Medical University and Academia Sinica, Taipei, Taiwan ROC.; 6 TMU Research Center of Cancer Translational Medicine, Taipei Medical University, Taipei, Taiwan ROC.; 7 Medical laboratory, Medical Education and Research Center, Kaohsiung Armed Forces General Hospital, Kaohsiung, Taiwan ROC.; 8 Division of Experimental Surgery Center, Department of Surgery, Tri-Service General Hospital, National Defense Medical University, Taipei, Taiwan ROC.; 9 Institute of Medical Science and Technology, National Sun Yat-sen University, Kaohsiung, Taiwan ROC.; 10 Department of Emergency Medicine, Kaohsiung Armed Forces General Hospital, Kaohsiung, Taiwan ROC.; 11 Department of Emergency Medicine, Tri-Service General Hospital, National Defense Medical University, Taipei, Taiwan ROC.; 12 Cancer Biology and Precision Therapeutics Center and Research Center for Cancer Biology, China Medical University, Taichung, Taiwan ROC.

**Keywords:** AMIGO2, pancreatic adenocarcinoma (PAAD), cell adhesion, EMT, migration, invasion

## Abstract

Pancreatic adenocarcinoma (PAAD), the predominant form of pancreatic cancer, is highly aggressive and refractory to current therapies. The Amphoterin-Induced Gene and ORF (AMIGO) family encodes three structurally related type I transmembrane proteins (AMIGO1-3) containing leucine-rich repeat and immunoglobulin-like domains, which mediate cell adhesion and signaling. Although AMIGO proteins have been implicated in neural development and tumor progression, their functional relevance in PAAD remains unclear. Here we identify AMIGO2 as a key driver of PAAD progression through integrated transcriptomic, proteomic, and functional analyses. Multi-cohort datasets (ONCOMINE, TCGA) and immunohistochemistry revealed marked AMIGO2 overexpression in PAAD tissues, with recurrent genetic alterations (~11%) and strong association with poor relapse-free and overall survival. Functional enrichment of AMIGO2-correlated genes indicated activation of focal adhesion and PI3K/AKT signaling. Consistently, inhibition of AMIGO2 expression in pancreatic cancer cells reduced migration and invasion while restoring E-cadherin expression, indicating inhibition of epithelial mesenchymal transition. Protein profiling from the Human Protein Atlas further confirmed elevated AMIGO2 expression in tumors. Together, these findings demonstrate that AMIGO2 promotes PAAD aggressiveness by enhancing adhesion- and EMT-associated pathways, establishing it as a potential prognostic biomarker and therapeutic target in pancreatic cancer.

## Introduction

Pancreatic cancer is one of the most lethal malignancies worldwide, with PAAD accounting for approximately 85% of all cases 1. PAAD is characterized by its aggressive nature, silent onset, and resistance to current therapies. Clinical symptoms such as abdominal pain, weight loss, and jaundice typically manifest only at advanced stages 2. Consequently, nearly 80-85% of patients are diagnosed when the disease is already unresectable or metastatic 3, 4. Although surgical resection remains the only curative treatment, only a small proportion of patients qualify for surgery due to late-stage diagnosis 5. The overall five-year survival rate remains dismal around 10%, making PAAD one of the deadliest solid tumors 3, 4.

In the absence of curative options, systemic chemotherapy constitutes the mainstay of treatment. However, intrinsic and acquired chemoresistance severely limit its efficacy 6, 7. The persistently poor survival rate largely attributable to late-stage detection, intrinsic drug resistance, and limited response to standard regimens underscores the urgent need for novel treatment strategies 8, 9. Despite numerous efforts to understand the molecular basis of PAAD and to develop new treatments, it remains a refractory malignancy 10. This highlights the importance of identifying novel oncogenic drivers that may contribute to treatment resistance and poor clinical outcomes. Epithelial mesenchymal transition (EMT), a central process that drives pancreatic cancer dissemination, chemoresistance, and immune evasion, represents a critical biological context for uncovering such regulators. Exploring such molecules could provide valuable insight into treatment failure and offer new targets for both diagnosis and therapy, especially for metastatic PAAD, which carries the worst prognosis. Therefore, there is critical to identify novel and reliable biomarkers for PAAD that can enable early diagnosis, predict clinical outcomes, and guide treatment decisions.

AMIGO1, AMIGO2 and AMIGO3 constitute a structurally similar family of type I transmembrane proteins, each containing six leucine-rich repeat (LRR) domains and one immunoglobulin (Ig)-like domain 11-13. These proteins are thought to be involved in cell-cell adhesion and signal transduction. AMIGO1 is predominantly expressed in the nervous system and plays a key role in neural development and myelination 14. AMIGO3, enriched in the brain, is also expressed in adult mouse tissues such as the liver, intestine, and lungs; its expression is rapidly upregulated after spinal cord injury, suggesting involvement in acute oligodendrocyte damage 15. AMIGO2, in contrast, is more broadly expressed across multiple tissues and has drawn increasing attention due to its emerging roles in cancer biology 16.

Structurally, AMIGO2 encodes a type I transmembrane protein consisting of a signal peptide for secretion, a transmembrane domain, six LRR motifs, and one immunoglobulin-like domain 14, 17. The LRR motifs are associated with protein-protein interactions on the cell surface and may participate in ligand binding and cell adhesion. Among the three members, AMIGO2 has received particular attention in cancer research 16, 18, 19. In colorectal cancer (CRC), elevated AMIGO2 expression has been linked to liver metastasis and poor prognosis. Mechanistically, AMIGO2 promotes epithelial-mesenchymal transition (EMT) by activating the TGF-β/Smad signaling pathway, thereby enhancing the invasive capacity of CRC cells. Notably, treatment with the TGF-β receptor inhibitor LY2109761 can suppress AMIGO2-induced EMT 16. Moreover, recent studies have highlighted the potential involvement of AMIGO2 in cancer progression 20. As the abnormal activation of the PI3K-AKT signaling pathway is a well-known trigger in many cancers 21-23, AMIGO2 has been suggested to participate in the transduction of this pathway, thereby regulating tumor growth and angiogenesis 24. For instance, in gastric cancer and pancreatic cancer, differential expression of AMIGO2 has been observed 17, 25. Moreover, AMIGO2 has been shown to enhance the adhesiveness of liver endothelial cells in colorectal and gastric cancers, thereby facilitating liver metastasis 17, 18, 26-29. Taken together, these findings suggest that AMIGO family proteins may play a significant role in cancer progression and metastasis. However, the role of AMIGO family proteins in PAAD remains largely unknown.

In this study, we conducted a comprehensive multi-omics analysis integrating transcriptomic, genomic, and immunohistochemical datasets, complemented by *in vitro* functional validation. By elucidating the expression patterns, prognostic significance, and biological functions of AMIGO2 in PAAD, particularly its potential involvement in EMT and cell motility. We identify AMIGO2 as a promising prognostic biomarker and therapeutic target, providing a foundation for improved diagnostic and precision-medicine strategies in pancreatic cancer.

## Materials and Methods

### ONCOMINE database analysis

The mRNA expression of AMIGO2 in various cancer types were analyzed by using Oncomine database (https://www.oncomine.org), an extensive repository of cancer microarray data 28. Student's t test was used to compare the transcription levels of AMIGO2 in Different Types of PAAD with that in normal controls. The thresholds were set for statistical significance at P < 0.01, and fold Change > 2.

### Gene expression profiling interactive analysis (GEPIA)2 database analysis

GEPIA2 (http://gepia2.cancer-pku.cn/) is a web-based interactive analysis tool designed for analyzing RNA sequencing expression data derived from The Cancer Genome Atlas (TCGA) and the Genotype-Tissue Expression (GTEx) databases. This platform consists of 9,736 tumor samples and 8,587 normal tissues, with all datasets processed through standardized computational pipelines, ensuring compatibility across the board 27, 30. This analytical platform was used to analyze AMIGO2 expressions in PAAD.

### Kaplan-Meier plotter

Kaplan-Meier Plotter (http://kmplot.com/), an online tool that enables the analysis of gene expression data in relation to survival in 35k+ samples from 21 tumor types. The database contains gene expression data and corresponding survival information from GEO, EGA, TCGA. The survival curves were further analyzed to calculate hazard ratios (HRs), 95% confidence intervals (CIs), and log-rank p-values 29. Our study used Kaplan-Meier Plotter to analysis the relapse-free survival (RFS) and overall survival (OS) of PAAD patients. Both RFS and OS curves showed the transcriptional expression of AMIGO1, AMIGO2, AMIGO3.

### cBioPortal analysis

cBioPortal (https://www.cbioportal.org/) is a comprehensive web platform that offers "gene-based" visualizations and analyses, providing crucial information related to cancer studies and genomic profiles. This tool is invaluable for researchers seeking to understand the genetic underpinnings of cancer, including the identification of altered genes and their associations with clinical outcomes 26. In our study, we utilized cBioPortal to analyze the genetic alterations and co-expression genes of the AMIGO family in PAAD using the TCGA PanCancer Atlas dataset. This study explores the genomic profiles of AMIGO1, AMIGO2, and AMIGO3 across 168 PAAD patients through cBioPortal platform.

### GeneMANIA analysis

GeneMANIA (http://genemania.org/) is an online analytical tool that facilitates the derivation of hypotheses based on gene functions 31. It enables users to query and generate a list of genes with similar functions to the target gene and visualizes the relationship between the target gene and the dataset by constructing an interactive network. The analysis provided insights into the gene-association network of AMIGOs.

### STRINGS analysis

STRING (https://string-db.org/) collects and integrates protein-protein interactions—both physical interactions as well as functional associations. The data originate from a number of sources: automated text mining of the scientific literature, computational interaction predictions from co-expression, conserved genomic context, databases of interaction experiments and known complexes/pathways from curated sources 32, 33. In this study, we used Protein-protein interaction (PPI) to conduct potential interactors between AMIGOs to explore the predicted.

### VENNY analysis

Venny (https://bioinfogp.cnb.csic.es/tools/venny) is a web-based tool designed for creating Venn diagrams, which are useful for visualizing the relationships between different sets of data, particularly in genomic fields where identifying common genes across multiple datasets is often required. is a web-based tool designed for creating Venn diagrams, which are useful for visualizing the relationships between different sets of data, particularly in genomic fields where identifying common genes across multiple datasets is often required 34. We identified the top 1000 genes which was related to AMIGOs in mRNA expression from cBioPortal to study the co-expression gene by using VENNY.

### DAVID

DAVID (https://david.ncifcrf.gov/) is a comprehensive bioinformatics resource that provides a suite of tools for functional annotation and enrichment analysis of gene lists. It operates by performing Gene Ontology (GO) enrichment analysis on user-submitted gene lists, with GO terms classified into three main categories: Biological Process (BP), Cellular Component (CC), and Molecular Function (MF). In our study we predict the functions of AMIGO2 these three basis aspects.

### Kyoto encyclopedia of genes and genomes (KEGG) signaling pathway analyses

KEGG is a comprehensive database resource for the biological interpretation of genomic sequences and other high-throughput data. It associates the molecular functions of genes and proteins with orthologous groups through the KEGG Orthology (KO) database 35. We used KEGG dataset to analyze the signaling pathway of AMIGO2. When P value < 0.05, the results were seen as significant. The false discovery rate (FDR) is typically set at a threshold of 0.05, meaning that a pathway is considered significantly enriched when the FDR is less than 0.05.

### The human protein atlas database

The Human Protein Atlas (HPA, https://www.proteinatlas.org/) is a comprehensive website dedicated to mapping all human proteins across cells, tissues, and organs by integrating various omics technologies. This includes antibody-based imaging, mass spectrometry-based proteomics, transcriptomics, and systems biology approaches. The HPA database comprises immunohistochemistry-based expression data for 17 major cancer types and 44 different tissue types 36. In our study, we confirmed AMIGO2 protein expression in PAAD and normal tissues by using HPA.

### Cell lines and cell culture conditions

The human pancreatic cancer cell line (MiaPaCa-2) was cultured in Dulbecco's Modified Eagle Medium (DMEM, high glucose; Gibco) containing 10% fetal bovine serum (FBS; Gibco), 100 U/mL penicillin (Gibco), 100 µg/mL streptomycin (Gibco), and 2 mM L-glutamine (Thermo), and maintained in a humidified incubator supplied with 5% CO₂ 37.

### shRNA knockdown and antibodies

The shRNA sequences targeting the AMIGO2 gene were purchased from the RNAi Core Facility at Academia Sinica (Taiwan). HEK293T cells were transfected with the AMIGO2 shRNA plasmids together with packaging plasmids following standard lentiviral production protocols. Transfected cells were cultured until lentiviral particles were produced. Viral supernatants were collected, filtered, nd used to infect MiaPaCa-2 cells in DMEM containing 10% fetal bovine serum (FBS), 100 U/mL penicillin, 100 µg/mL streptomycin, and 2 mM L-glutamine, in a 37 °C incubator supplied with 5% CO₂. After assessing infection efficiency, lentiviruses carrying control shRNA (shCTL) or AMIGO2 shRNAs (shAMIGO2-A1 and shAMIGO2-B2) were used to infect MiaPaCa-2 cells for 72 h. Media containing residual viral particles were replaced with fresh complete medium before further assays. Primary antibodies used in this study included: AMIGO2 (E-AB-10790, Elabscience), E-cadherin (R55-180, BD Biosciences), Vimentin (E-AB-22085, Elabscience), and α-Tubulin (T6199, Sigma-Aldrich). Secondary antibodies included anti-rabbit IgG (GeneTex) and anti-mouse IgG (GeneTex).

### Wound healing assay

Cell migration was evaluated using a wound-healing assay. MiaPaCa-2 cells were seeded in 6-well plates and grown to 90-100% confluence in complete medium. A sterile 200 µL pipette tip was used to create a linear scratch across the cell monolayer. The wells were gently washed with PBS to remove detached cells and then incubated in serum-free DMEM at 37 °C with 5% CO₂. Wound closure was monitored and imaged at 0, 24, and 48 h using a phase-contrast microscope. The wound area was quantified using ImageJ software and expressed as the percentage of wound closure relative to the initial wound area. All experiments were performed in triplicate.

### Transwell assay

Cell invasion ability was assessed using a Transwell assay. MiaPaCa-2 cells (2 × 10⁴ cells/well) were resuspended in serum-free medium and seeded into the upper chamber of 8-µm pore Transwell inserts (Corning) pre-coated with Matrigel (BD Biosciences) 38. The lower chamber was filled with DMEM containing 10% FBS as a chemoattractant. After incubation at 37 °C for 24 h, non-invading cells on the upper surface were gently removed with a cotton swab. Cells that invaded to the lower surface were fixed with 4% paraformaldehyde (PFA) for 15 min, stained with 0.1% crystal violet for 20 min, and imaged under an inverted microscope. The invaded cell area was quantified from five random fields per insert using ImageJ, and all experiments were conducted in triplicate.

### Statistics

All experiments were performed in triplicate, and data are presented as mean ± standard deviation (SD). Statistical analyses were carried out using GraphPad Prism 9. Differences between two groups were evaluated using the unpaired Student's *t*-test, and comparisons among multiple groups were analyzed using one-way ANOVA followed by Tukey's post hoc test 39. A *p*-value of < 0.05 was considered statistically significant.

## Results

### Expression patterns of AMIGOs in PAAD patients

**Figure 1:**
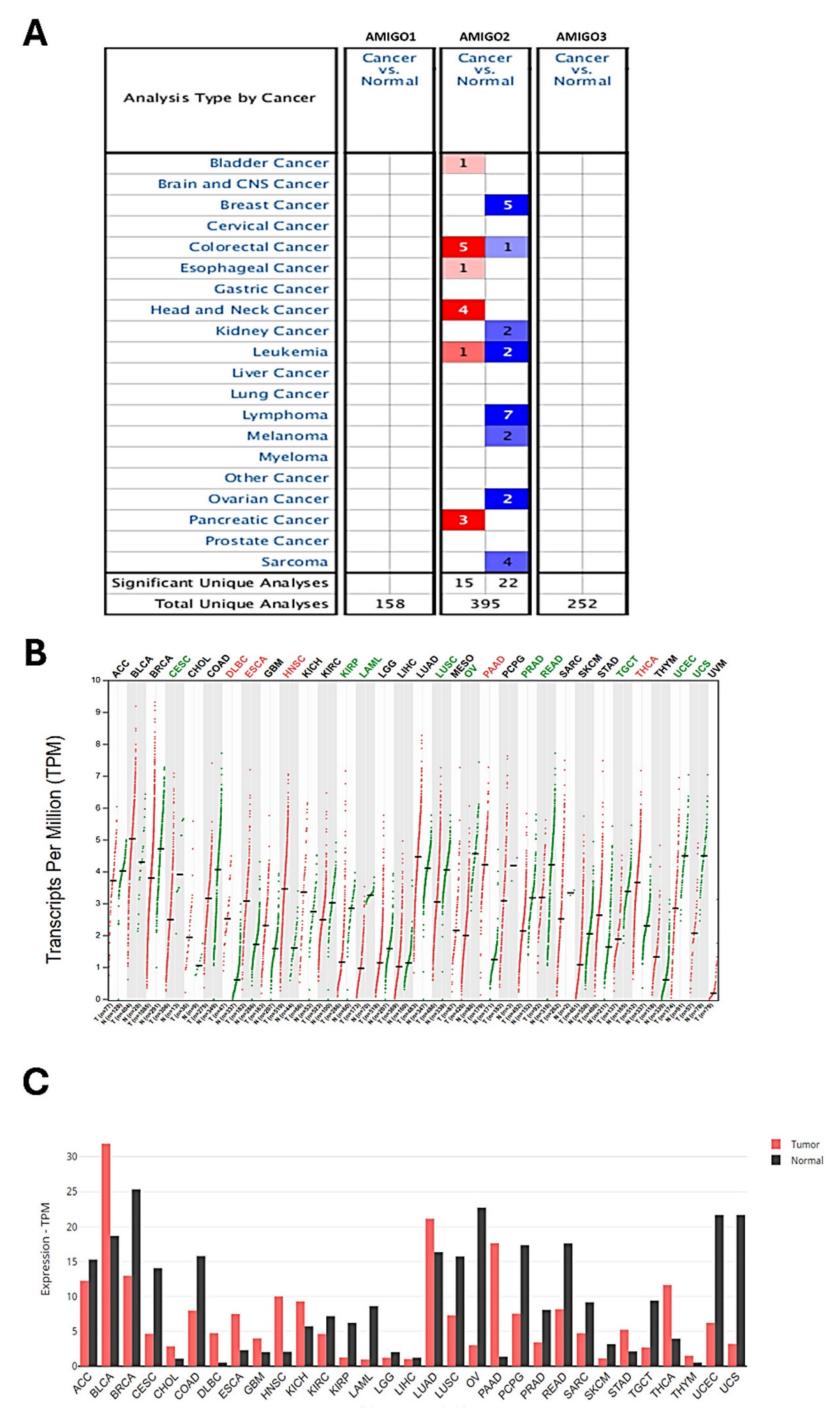
** Transcriptional expression of AMIGO family members in various cancer types.** (A) Expression profile of AMIGO1, AMIGO2 and AMIGO3 across different cancers analyzed using the ONCOMINE database. (B, C) Differential mRNA expression of AMIGO2 in pancreatic adenocarcinoma (PAAD) compared to normal pancreatic tissues, analyzed using GEPIA2.

To investigate whether AMIGO family proteins are differentially expressed in PAAD, we used the Oncomine database to compare the mRNA expression of AMIGO2 in different cancer and normal tissue samples. ONCOMINE analysis showed that AMIGO2 was abnormally expressed in most of the tissue samples, and it also showed the transcriptional levels of AMIGO2 were upregulated in PAAD patients **(Figure 1A)**. Within three PAAD datasets analysis of ONCOMINE, the transcription level of AMIGO2 in PAAD was significantly higher than normal tissues, with fold changes of AMIGO2 of 3.763 (P = 6.26E-14), 2.695 (P = 6.99E-5) and 3.421 (P = 2.42E-7), respectively (Table 1). In contrast, no significant difference was observed for AMIGO1 and AMIGO3 in PAAD datasets. Next, we further compared the transcriptional expression of AMIGO2 in PAAD and normal tissues in the GEPIA2 database. Through the differential gene expression analysis across all tumor samples and paired normal tissues, we found out AMIGO2 significantly expressed in PAAD **(Figure 1B and 1C)** and was 12 times higher than normal tissue (median expression of tumor sample = 17.64, median expression of normal tissue = 1.37) **(Table 1)**.

### The transcription level of AMIGOs and the clinicopathological parameters in PAAD patients

**Figure 2:**
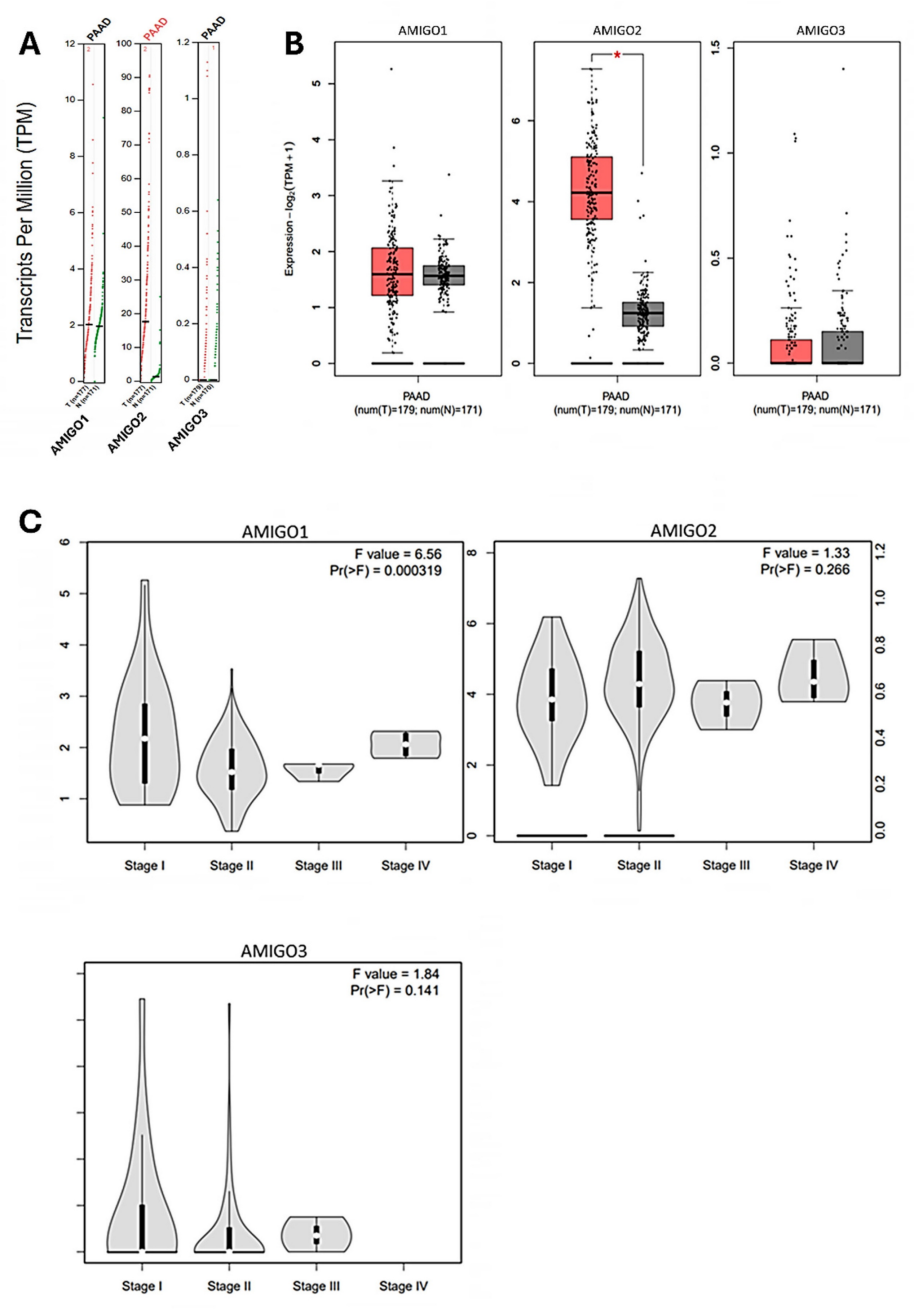
** Transcriptional expression and pathological stage analysis of AMIGO family members in PAAD using GEPIA2 database.** (A-B) Comparison of AMIGO1, AMIGO2 and AMIGO3 mRNA expression levels between PAAD and normal pancreatic tissues. (C) Expression levels of AMIGOs across different tumor stages in PAAD patients.

To further investigate the clinical significance of AMIGO family members in PAAD, we examined their transcriptional expression profiles in relation to tumor tissues and pathological stages. Utilizing the GEPIA2 platform, we compared the tissue transcriptional expression levels of AMIGO1, AMIGO2 and AMIGO3 between PAAD and normal pancreatic tissues **(Figure 2A and 2B)**. In addition, we explored the expression patterns of AMIGO family members across different pathological stages of PAAD. The results indicated that AMIGO1 expression was significantly associated with tumor stage, suggesting its potential role in the progression of PAAD **(Figure 2C)**. In contrast, AMIGO2 and AMIGO3 did not show significant stage-specific expression patterns. These findings highlight the differential expression profiles of AMIGO family members in PAAD and underscore the potential clinical relevance of AMIGO1 in disease progression. In sum, our analysis revealed that AMIGO2 was significantly upregulated in PAAD tissues compared to normal tissues, whereas AMIGO1 and AMIGO3 did not exhibit statistically significant differences in expression.

### The prognosis value between mRNA expression of AMIGOs in PAAD patients

**Figure 3:**
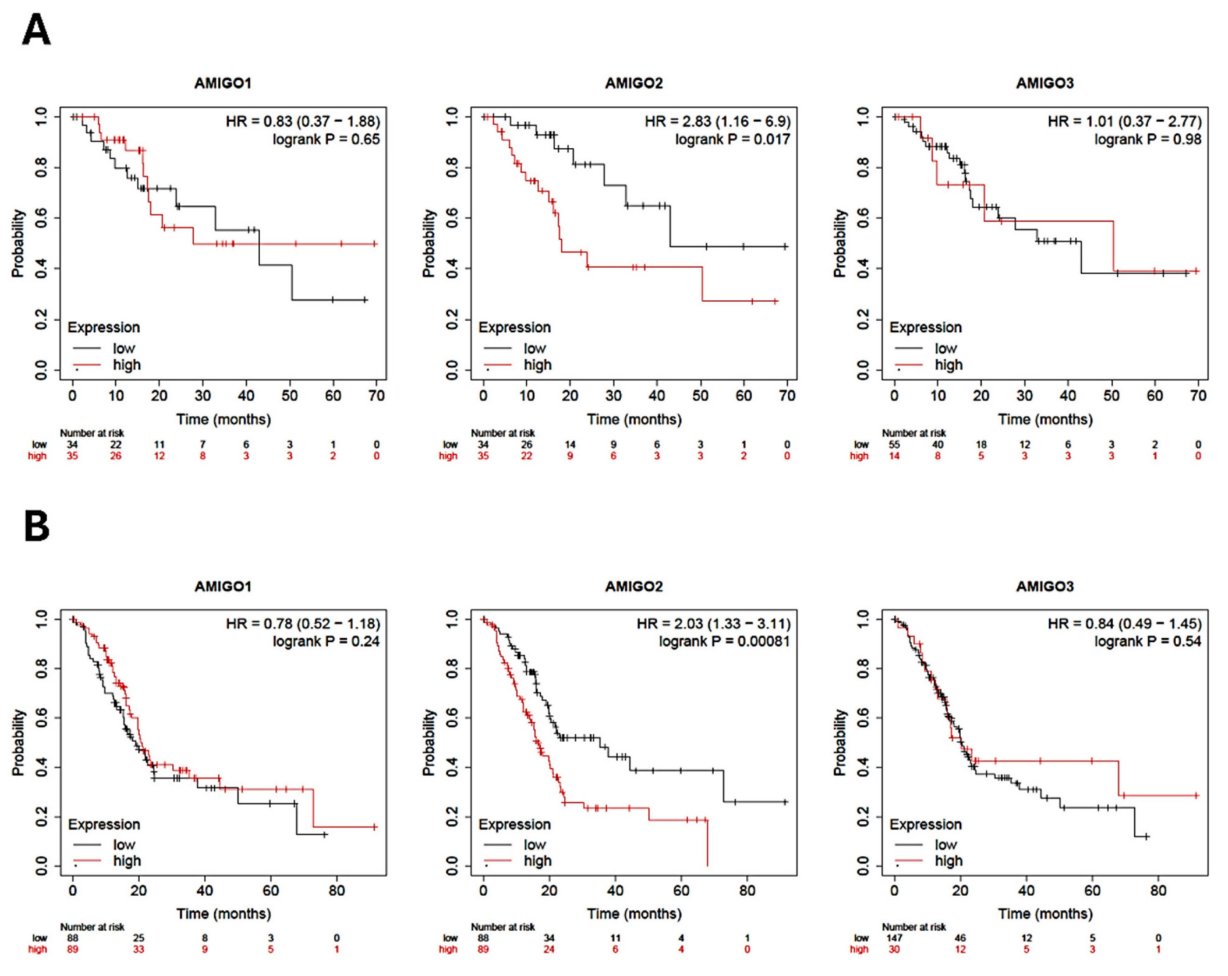
** Prognostic value of AMIGO family in pancreatic adenocarcinoma (PAAD) patients.** (A) Relapse-free survival (RFS) curves and (B) Overall survival (OS) of AMIGO1, AMIGO2 and AMIGO3 in PAAD.

To comprehensively assess the clinical significance of AMIGO2 in PAAD, we conducted a detailed survival analysis using the Kaplan-Meier Plotter, focusing on both relapse-free survival (RFS) and overall survival (OS) in patients diagnosed with PAAD. Our analysis revealed that the high transcriptional expression levels of AMIGO2 are significantly associated with poor patient survival outcomes **(Figure 3A and 3B)**. Both RFS and OS curves showed that the transcriptional expression of AMIGO2 was significantly related to survival time (P = 0.017 and P = 0.00081). In the Kaplan-Meier Plotter dataset, the hazard ratios (HR) of AMIGO1 are 0.83(P = 0.65) for RFS and 0.78 (P = 0.24) for OS, respectively. As for AMIGO2, they are 2.83(P = 0.017) and 2.03 (P = 0.00081) for RFS and OS, respectively. For AMIGO3, they are 1.01 (P = 0.98) and 0.84 (P = 0.54) for RFS and OS, respectively. A pan-cancer survival comparison using TCGA data further demonstrated that the prognostic impact of AMIGO2 was predominantly observed in PAAD (HR = 2.21, p = 0.00045), whereas no significant associations were detected in COAD, STAD, BRCA, or LUAD **(Supplementary Figure S1 and Table S1)**. Collectively, these findings demonstrate that AMIGO2 serves as a robust prognostic indicator in PAAD, with its elevated expression specifically associated with unfavorable clinical outcomes compared with other AMIGO family members or cancer types.

### Comprehensive genomic and interaction network analysis of AMIGO family members in PAAD

**Figure 4:**
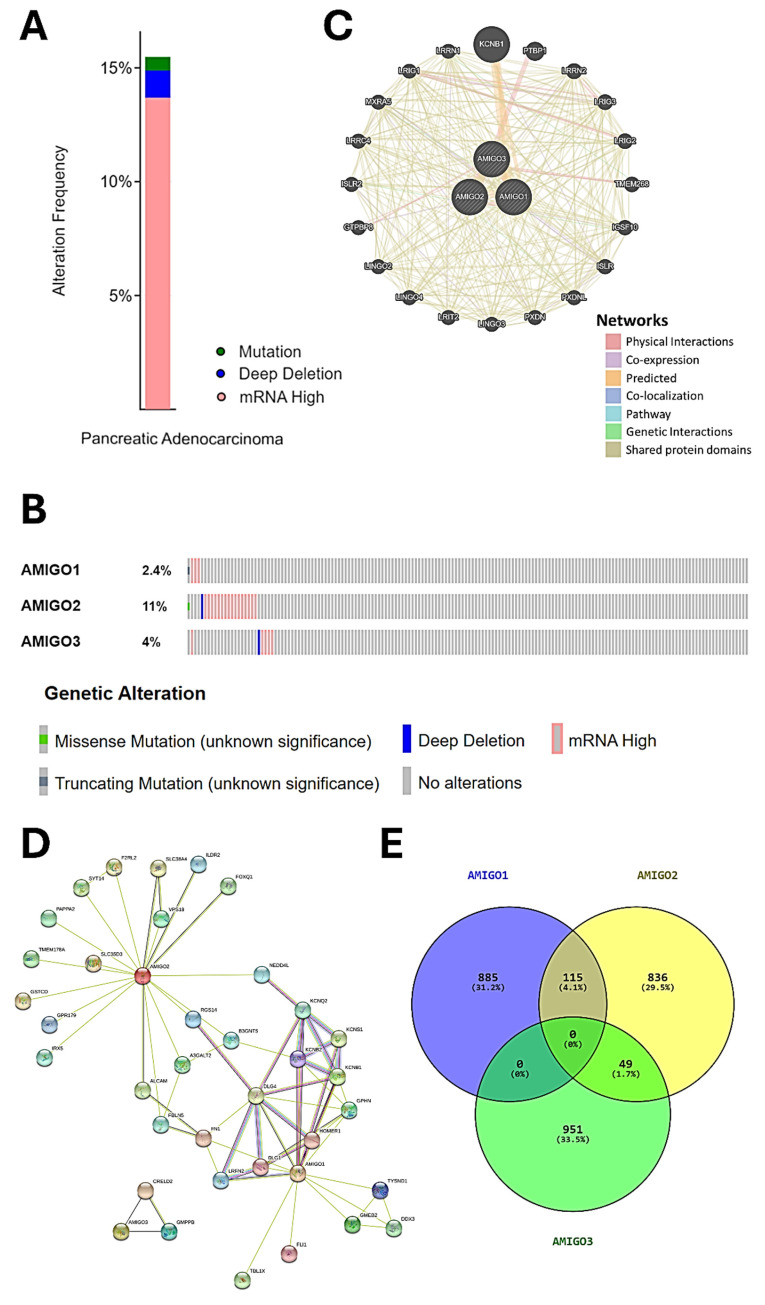
** Genetic alterations, gene and protein interaction networks and co-expression analysis of AMIGO family members in pancreatic adenocarcinoma (PAAD).** (A-B) Genetic alteration analysis of AMIGO1, AMIGO2 and AMIGO3 in PAAD patients using cBioPortal. (C) Gene-gene interaction (GGI) network of AMIGOs constructed by GeneMANIA. (D) Protein-protein interaction (PPI) network of AMIGOs generated using the STRING database. (E) Co-expression gene analysis of AMIGOs based on the intersection of top 1000 correlated genes, visualized using VENNY.

We next accessed the cBioPortal database to analyze the genetic alterations of the AMIGO family in total of 168 PAAD patients by using the TCGA PanCancer Atlas dataset. The genetic alterations in the AMIGO family included missense mutations, truncating mutations, deep deletions and high mRNA expression levels **(Figure 4A, 4B)**. The overall genetic alteration rate of the AMIGO family was 15.48% (26/168). Specifically, AMIGO2 exhibited the highest alteration rate at 11%, followed by AMIGO3 at 4% and AMIGO1 at 2.4%. These findings indicate that genetic alterations in the AMIGO family, particularly in AMIGO2, occur at a notable frequency in PAAD patients.

Furthermore, the network of gene-gene interaction (GGI) can predict the function between AMIGOs by using GeneMANIA database **(Figure 4C)**. There were 20 genes strongly related to AMIGOs in physical interactions (77.64%), co-expression (8.01%), predicted (5.37%), co-localization (3.69%), genetic interactions (2.87%), pathway (1.88%) and shared protein domains (0.6%). Protein-protein interaction (PPI) can study the potential interactors between AMIGOs to explore the predicted interaction by using STRING platform **(Figure 4D)**. The number of nodes was 38, the number of edges was 69, and the expected number of edges was 42. It showed the network had significantly more interactions than expected (PPI enrichment p-value = 9.87e-05). Moreover, we further identified the top 1000 genes which were related to AMIGOs in mRNA expression from cBioPortal to study the co-expression gene by using VENNY analysis **(Figure 4E)**. The result showed that **no gene was co-expressed among all three AMIGO family members simultaneously**, suggesting that each AMIGO may participate in distinct regulatory networks in PAAD. In addition, a combined alteration analysis of AMIGO family members (AMIGO1, AMIGO2, and AMIGO3) showed no significant difference in overall survival between altered and unaltered groups (Log-rank p = 0.855), indicating that AMIGO2 is the major prognostic determinant among the AMIGO family **(Supplementary Figure S2)**. This finding further supports that even when analyzed collectively, alterations in AMIGO1/2/3 fail to stratify patient outcomes, underscoring AMIGO2 as the principal clinically relevant member. Taken together, the abnormal expression of AMIGO2 is considered to be one of the oncogenes in PAAD.

### Functional enrichment and signaling pathway analysis of AMIGO2 in PAAD patients

**Figure 5:**
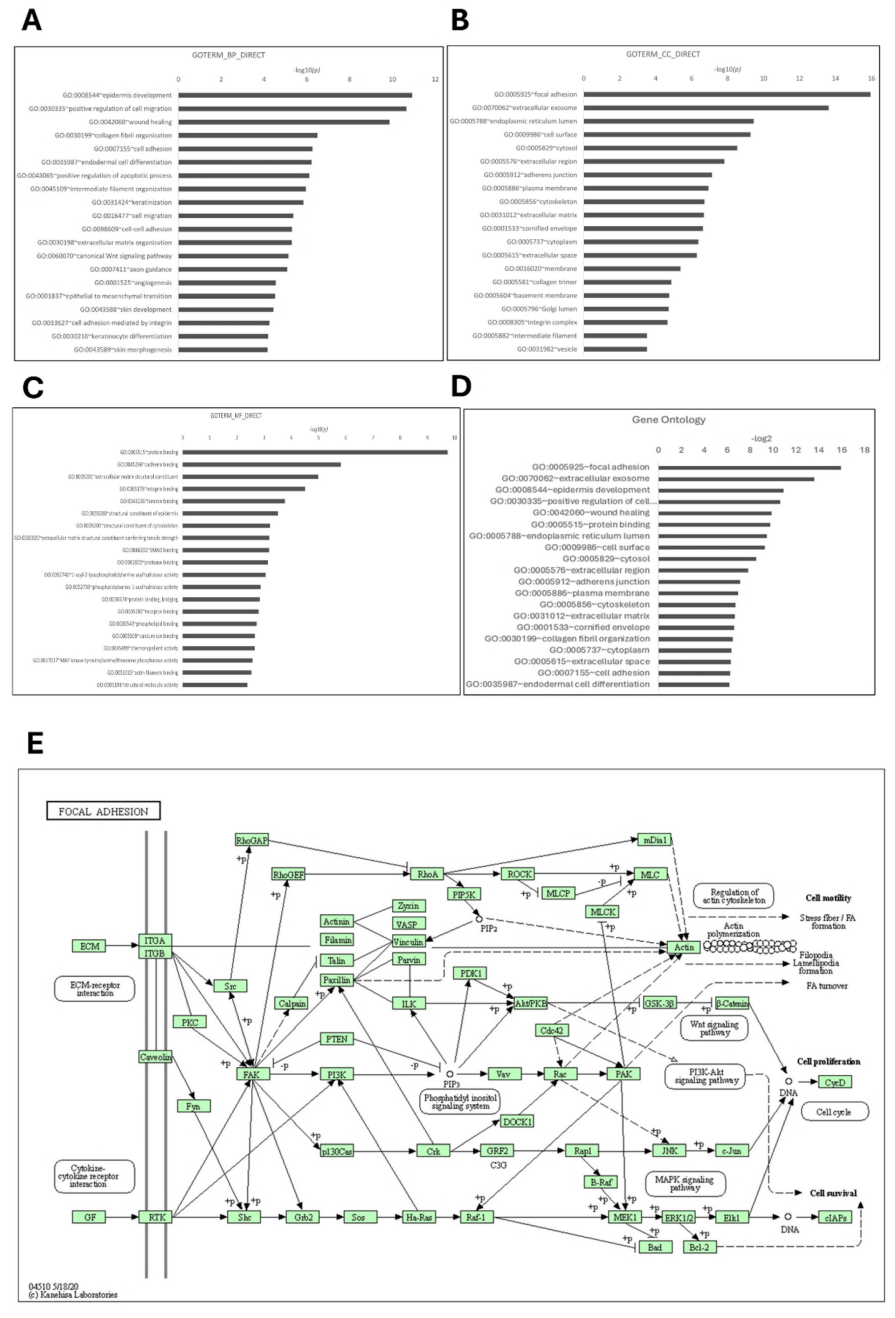
** Functional enrichment analysis of AMIGO2 in pancreatic adenocarcinoma (PAAD) by using the DAVID platform.** (A-C) Gene Ontology (GO) enrichment analysis of AMIGO2-related genes, categorized into biological processes (BP), cellular components (CC), and molecular functions (MF), respectively. (D) Top 20 GO terms enriched in AMIGO2 co-expressed genes. (E) Kyoto Encyclopedia of Genes and Genomes (KEGG) pathway analysis of AMIGO2-associated genes.

To investigate the biological functions associated with the AMIGO family in PAAD, we performed functional enrichment analysis using DAVID, focusing on genes co-expressed with AMIGO2, the most dysregulated member of the family. Gene Ontology (GO) classification revealed distinct enrichment patterns across the three major GO categories. In the Biological Process (BP) category, the top enriched terms included epidermis development (-log₁₀(p) = 11.00), positive regulation of cell migration (10.33), and wound healing (9.85) **(Figure 5A),** all of which are closely associated with tumor invasion, tissue remodeling, and metastatic behavior. In the Cellular Component (CC) category, focal adhesion and extracellular exosome were the most significantly enriched terms, with -log₁₀(p) values of 15.95 and 13.62, respectively **(Figure 5B)**, indicating a strong involvement in cell-matrix adhesion and extracellular signaling. For Molecular Function (MF), protein binding (-log₁₀(p) = 9.70) and cadherin binding (6.80) were significantly enriched **(Figure 5C)**, consistent with structural of AMIGO2 domains and its potential to mediate protein-protein and cell-cell interactions.

To further consolidate these findings, we summarized the top 20 GO terms across all categories **(Figure 5D)**. Consistent with the GO enrichment outcomes, KEGG pathway analysis revealed focal adhesion as the most significantly enriched pathway (*p* = 9.2 × 10⁻¹⁰), underscoring its potential role in cancer cell adhesion and metastasis **(Figure 5E).** These pathway-level findings may also help explain the poor clinical outcomes commonly observed in PAAD, where enhanced cell motility and adhesion facilitate early metastasis and resistance to treatment. Cumulatively, these enrichment results highlight that AMIGO2-associated genes are functionally linked to adhesion, migration, and tumor microenvironment interactions pathways that are intimately involved in the initiation and progression of metastasis **(Table 2)**. This functional profile provides a mechanistic basis for the metastatic behavior previously reported for AMIGO2 in other cancers and supports its potential role in driving tumor dissemination in PAAD.

### Protein expression and association of AMIGO2 with EMT and immune infiltration in PAAD

**Figure 6:**
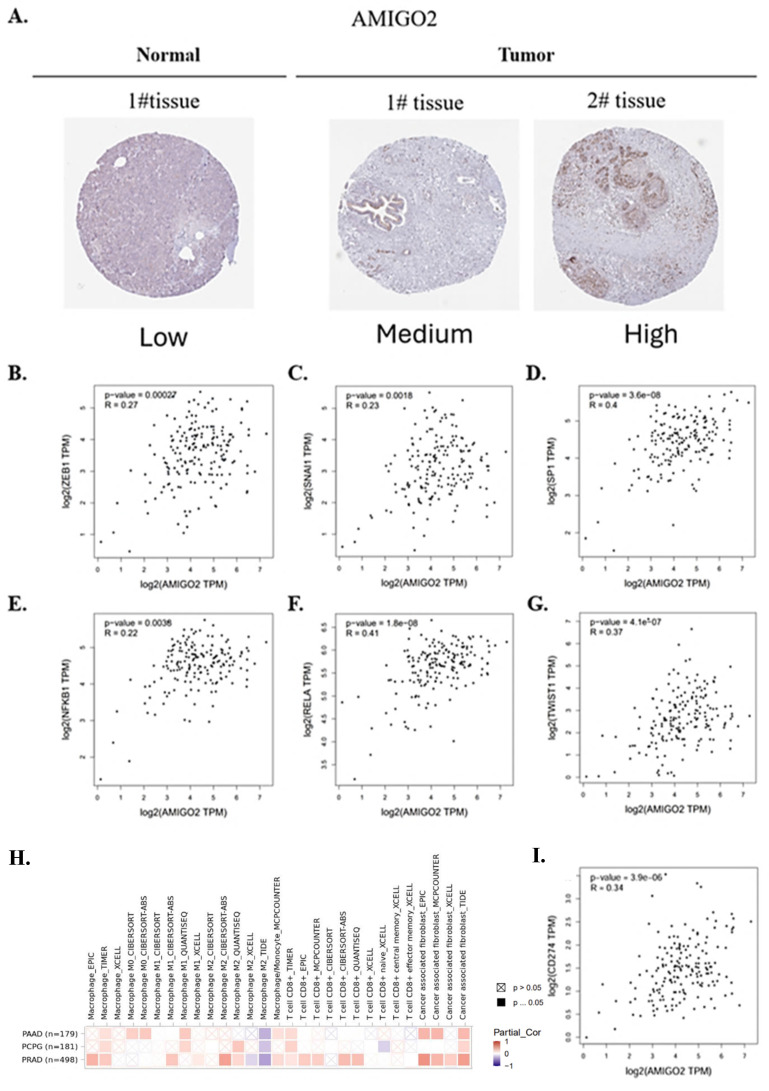
** Correlation between AMIGO2 and EMT-related transcription factors in PAAD.** (A) Protein expression of AMIGO2 in normal pancreatic tissues and PAAD tissues based on immunohistochemistry (IHC) images from the Human Protein Atlas (HPA). Representative IHC staining shows weak or absent AMIGO2 expression in normal pancreatic tissues (left) and stronger staining intensity in PAAD tissues (right). (B-G) Correlation analyses between AMIGO2 and key EMT-related transcription factors in TCGA-PAAD. Scatter plots show the Spearman correlation between AMIGO2 expression and ZEB1 (ρ = 0.27, p = 2.7 × 10⁻⁴), SNAI1 (ρ = 0.23, p = 1.8 × 10⁻³), SP1 (ρ = 0.40, p = 3.6 × 10⁻⁸), NFKB1 (ρ = 0.22, p = 3.8 × 10⁻³), RELA (ρ = 0.41, p = 1.8 × 10⁻⁸) and TWIST1 (ρ = 0.37, p = 4.1 × 10⁻⁷). (H) Correlation between AMIGO2 expression and immune cell infiltration across TCGA cancer types, including pancreatic adenocarcinoma (PAAD), pheochromocytoma and paraganglioma (PCPG), and prostate adenocarcinoma (PRAD), analyzed using TIMER2.0 with tumor purity adjustment. The heatmap displays partial Spearman correlation coefficients across multiple immune deconvolution algorithms. (I) Correlation between AMIGO2 and CD274 (PD-L1) expression in TCGA-PAAD, analyzed using GEPIA2 (Spearman correlation). Scatter plot shows a significant positive correlation (R = 0.34, *p* = 3.9 × 10⁻⁶), indicating that AMIGO2-high tumors exhibit elevated PD-L1 expression.

To further validate the protein expression of AMIGO2 and explore its potential biological relevance in PAAD, we conducted integrative analyses combining immunohistochemistry (IHC), EMT-related transcription factor correlation, and immune infiltration profiling. As shown in **Figure 6A**, IHC images from the Human Protein Atlas (HPA) demonstrated markedly elevated AMIGO2 protein expression in PAAD tissues compared with normal pancreatic tissues. Strong cytoplasmic and membranous staining was observed in tumor cells, whereas normal pancreatic tissues exhibited minimal or no detectable staining. To assess the association between AMIGO2 and epithelial-mesenchymal transition (EMT), correlation analyses using TCGA-PAAD data revealed that AMIGO2 expression was positively correlated with several EMT-related transcription factors, including ZEB1, SNAI1, SP1, NFKB1, RELA, and TWIST1 (**Figure 6B-G**). These findings suggest that AMIGO2 expression is correlated to EMT transcriptional activation, consistent with a pro-metastatic phenotype.

Furthermore, immune infiltration analysis using TIMER2.0 with tumor purity adjustment showed that AMIGO2 expression was positively correlated with macrophage and cancer-associated fibroblast (CAF) infiltration, while being negatively correlated with CD8⁺ T cell infiltration across several deconvolution algorithms (**Figure 6H**). These results indicate that AMIGO2 is associated with stromal remodeling and an immune-excluded tumor microenvironment in PAAD. Furthermore, AMIGO2 expression was positively correlated with CD274 (PD-L1) expression in TCGA-PAAD (R = 0.34, p = 3.9 × 10⁻⁶), suggesting that AMIGO2-high tumors may exhibit enhanced immune checkpoint activation and immune-evasive potential. Moreover, AMIGO2 expression positively correlates with CD274 (PD-L1) in PAAD, suggesting a possible link to immune-evasive tumor microenvironments and checkpoint activation **(Figure 6I)**. Collectively, these data demonstrate that AMIGO2 is overexpressed at the protein level and closely associated with both EMT activation and immune exclusion, suggesting its multifaceted role in PAAD progression.

### Inhibition of AMIGO2 suppresses EMT-associated migration and invasion in pancreatic cancer cells

**Figure 7:**
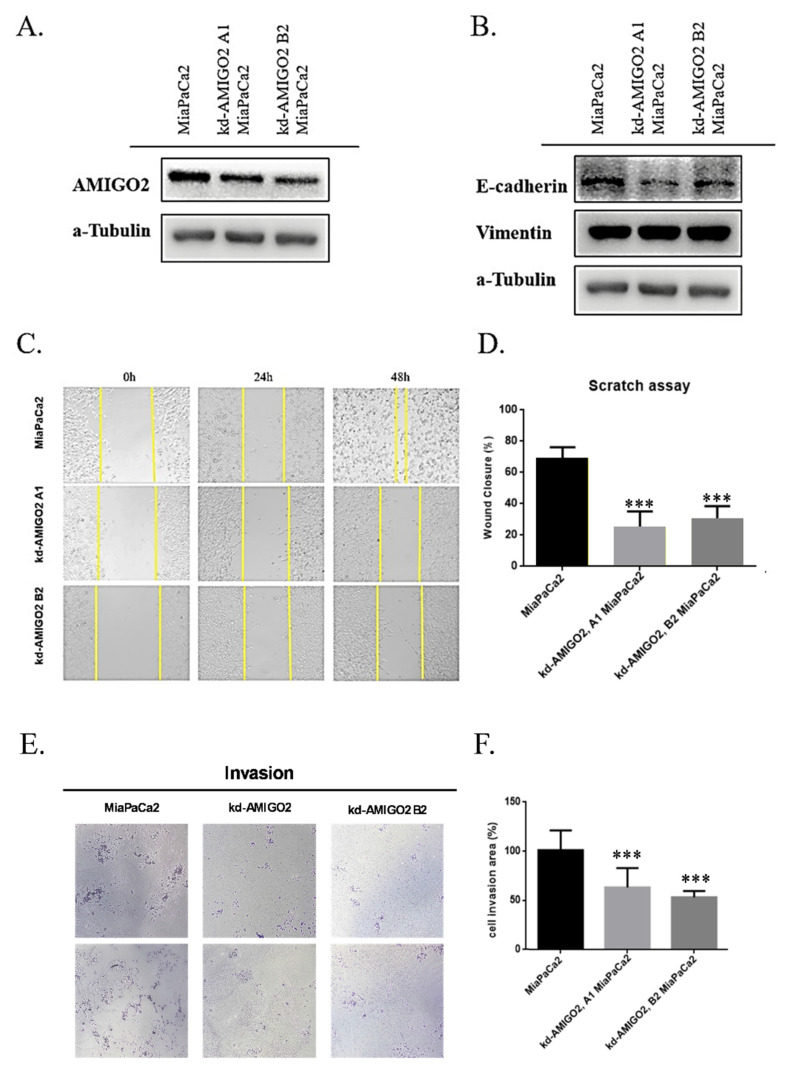
** AMIGO2 promotes migration and invasion by inducing EMT in pancreatic cancer cells.** (A) Western blot analysis of AMIGO2 protein levels in MiaPaCa-2 cells transfected with two independent AMIGO2 shRNAs (kd-AMIGO2 A1 and kd-AMIGO2 B2) compared with control cells. α-Tubulin served as a loading control. (B) Western blot analysis of EMT markers showing that AMIGO2 knockdown upregulated E-cadherin and downregulated Vimentin expression. α-Tubulin was used as an internal control. (C) Representative images of wound-healing assays performed in MiaPaCa-2 cells after AMIGO2 knockdown, captured at 0, 24, and 48 h. (D) Quantitative analysis of wound closure rates (%) in MiaPaCa-2 control and AMIGO2-silenced cells. (E) Representative images of Transwell invasion assays showing a reduced number of invading cells following AMIGO2 knockdown. (F) Statistical quantification of invaded cell area (%) based on Transwell assays. ***, p<0.01.

To functionally validate the role of AMIGO2 in PAAD, stable AMIGO2-knockdown MiaPaCa-2 cells were established using two independent shRNAs (kd-AMIGO2 A1 and kd-AMIGO2 B2). Western blot analysis confirmed a substantial reduction of AMIGO2 protein expression in both knockdown clones compared with control cells **(Figure 7A)**. Silencing AMIGO2 led to specific increase in E-cadherin expression indicating partial inhibition of EMT in pancreatic cancer cells **(Figure 7B)**. Functionally, AMIGO2 depletion markedly impaired the migratory ability of MiaPaCa-2 cells, as shown by the wound-healing assay, in which wound closure rates were significantly reduced relative to control cells **(Figure 7C-D)**. Similarly, Transwell invasion assays demonstrated that AMIGO2-knockdown cells exhibited a pronounced decrease in invasive capacity **(Figure 7E-F)**. Together, these findings provide direct experimental evidence that AMIGO2 functions as an active driver of EMT-associated migration and invasion in pancreatic cancer cells, thereby reinforcing its central role in PAAD metastasis.

## Discussion

In this study, we systematically investigated the role of AMIGO2 in PAAD by integrating multi-omics public datasets with in vitro validation. AMIGO2 was significantly overexpressed in PAAD compared with normal pancreatic tissues, as confirmed by ONCOMINE and GEPIA2 analyses. Elevated AMIGO2 was consistently associated with shorter overall and relapse-free survival, reinforcing its role as a negative prognostic indicator in PAAD. This observation aligns with recent efforts to identify both protein-coding and non-coding transcripts as clinically relevant biomarkers in PAAD 40. Furthermore, cBioPortal analyses showed that AMIGO2 was the most frequently altered AMIGO family member in PAAD, with alterations in ~11% of cases.

Functional enrichment using GO and KEGG revealed that AMIGO2-associated genes are involved in cell adhesion, extracellular vesicle/exosome biology, and most prominently the focal adhesion pathway. These results suggest that AMIGO2 may contribute to tumor cell adhesion, motility, and metastatic dissemination. Prior studies indicate that AMIGO2 encodes a type I transmembrane protein bearing extracellular LRRs that facilitate protein-protein interactions and ligand binding at the cell surface 14, 17, which may underlie its role in tumor cell-cell/cell-matrix adhesion. Similar adhesion-linked mechanisms have been reported for Cadherin-23 (CDH23), which correlates with poor prognosis in PAAD and promotes cell viability under non-adherent conditions 41. Given that focal adhesion complexes integrate cytoskeletal remodeling and motility signaling to initiate metastatic dissemination, and that AMIGO2 enhances hepatic endothelial adhesion and liver metastasis in gastric and colorectal cancers 18, 42-45, our enrichment data in PAAD extend this metastasis-associated role of AMIGO2.

Our enrichment results particularly those involving focal adhesion and cell-migration terms converge with prior reports linking AMIGO2 to metastatic progression and poor outcomes in gastrointestinal and pancreatic malignancies 20, 43, 46. In contrast, AMIGO1 and AMIGO3 have not been consistently associated with tumor aggressiveness or adverse prognosis in these settings and do not exhibit robust expression changes in PAAD. Together, these findings support the view that although AMIGO family members may broadly modulate tumor biology, AMIGO2 is the dominant functional driver in pancreatic adenocarcinoma, likely through adhesion-related mechanisms.

To experimentally validate these in silico findings, we next performed in vitro functional assays, which demonstrated that AMIGO2 plays an active functional role in promoting EMT and invasive behavior in pancreatic cancer cells. These results provide direct mechanistic evidence that AMIGO2 facilitates metastasis-related phenotypes, supporting its role as a driver of adhesion-dependent cell motility rather than a passive biomarker. Consistent with the focal-adhesion and PI3K/AKT pathway enrichment observed in our analyses, AMIGO2 may enhance cytoskeletal remodeling and intracellular signaling that collectively promote metastatic dissemination in PAAD.

Similar EMT-suppressive effects upon AMIGO2 silencing have recently been reported in other malignancies, including melanoma and gastric cancer 20, 42, while comparable EMT-inhibitory outcomes were also observed in bladder cancer following AMIGO2 knockdown 47. Collectively, these findings suggest that AMIGO2 may act as a conserved regulator of adhesion- and migration-related signaling across diverse tumor types. In human PAAD tissues, elevated EMT-related focal adhesion kinase (FAK) activity correlates with increased fibrosis and reduced CD8⁺ T-cell infiltration. Preclinical studies have shown that FAK inhibition alleviates stromal stiffness and enhances T-cell penetration into the tumor core 48. These findings suggest that AMIGO2 may influence cytotoxic T-cell activity through a FAK-mediated EMT mechanism of immune exclusion in PAAD 49.

## Conclusion

In summary, this study provides integrative and experimental evidence that AMIGO2 serves as a clinically and biologically relevant driver in PAAD. AMIGO2 was markedly overexpressed in tumor tissues, and its elevated expression correlated with poor overall and relapse-free survival. Among the AMIGO family, AMIGO2 showed the highest alteration frequency and strong enrichment in focal adhesion and cell migration pathways. Consistent with these bioinformatic findings, *in vitro* knockdown of AMIGO2 significantly impaired the migratory and invasive abilities of pancreatic cancer cells and upregulated E-cadherin expression, confirming its functional involvement in EMT and metastasis. Together, these results establish AMIGO2 as a key mediator of adhesion dependent tumor progression and highlight its potential as a prognostic biomarker and therapeutic target in PAAD.

## Supplementary Material

Supplementary figures and table.

## Figures and Tables

**Table 1:**
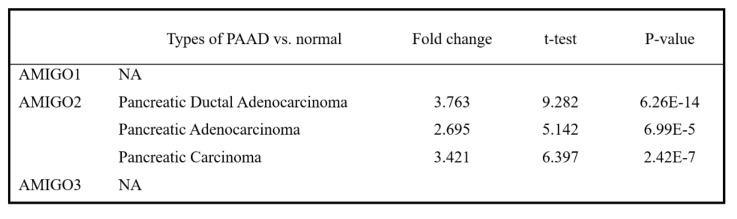
Differential mRNA expression of AMIGO family members in PAAD subtypes compared with normal tissues.

**Table 2:**
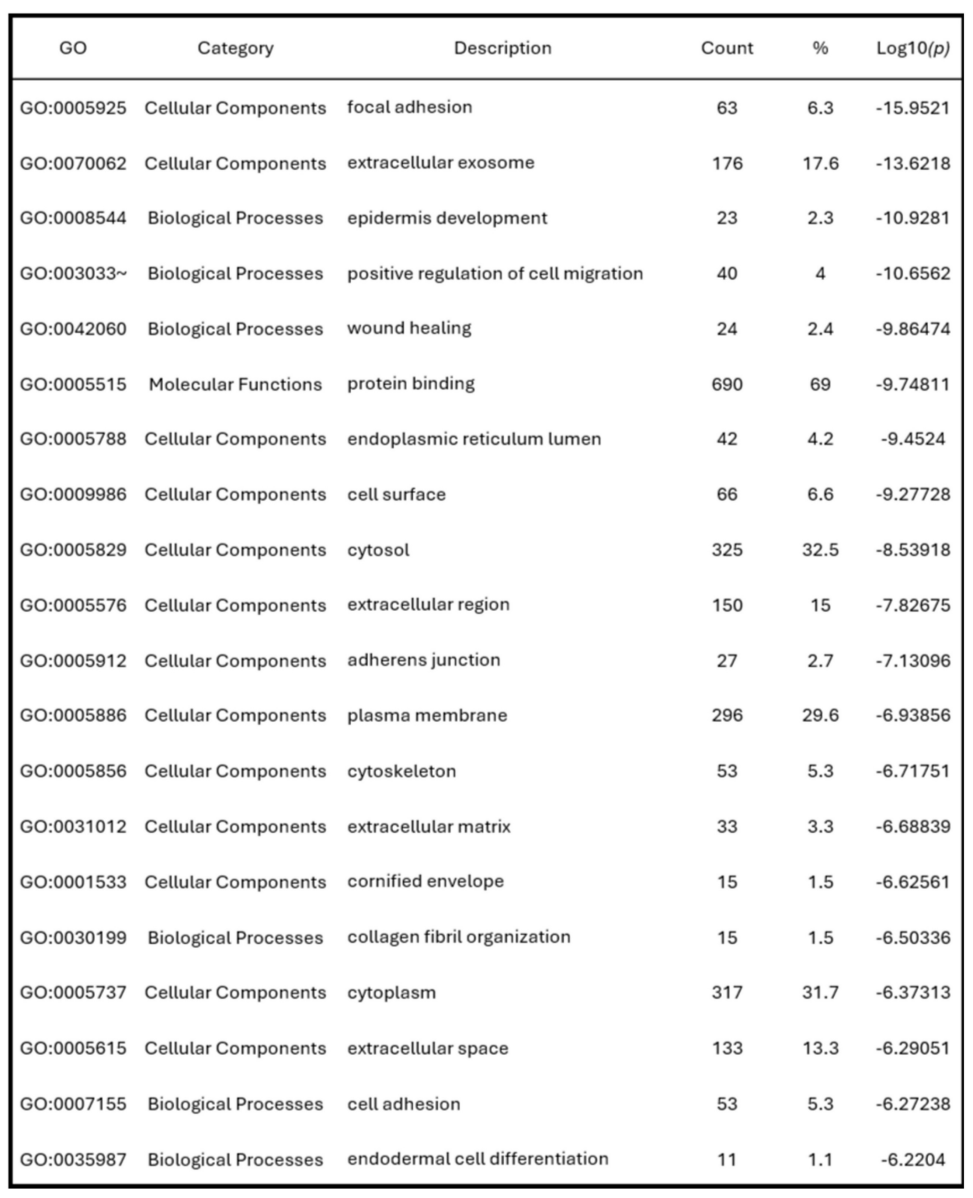
The top 20 GO functional enrichment analysis of AMIGO2.
