# Supplementary material for: AMIGO2 as a Novel Biomarker Predicting Poor Prognosis and Associated with Adhesion-Driven Metastasis in Pancreatic Adenocarcinoma
Source: Int J Med Sci. 2026 Jan 14;23(2):695–710. doi: 10.7150/ijms.121794 (PMC12825146; doi:10.7150/ijms.121794)
Supplement: Supplementary file 1 — Supplementary figures and table. [file ijmsv23p0695s1.pdf]

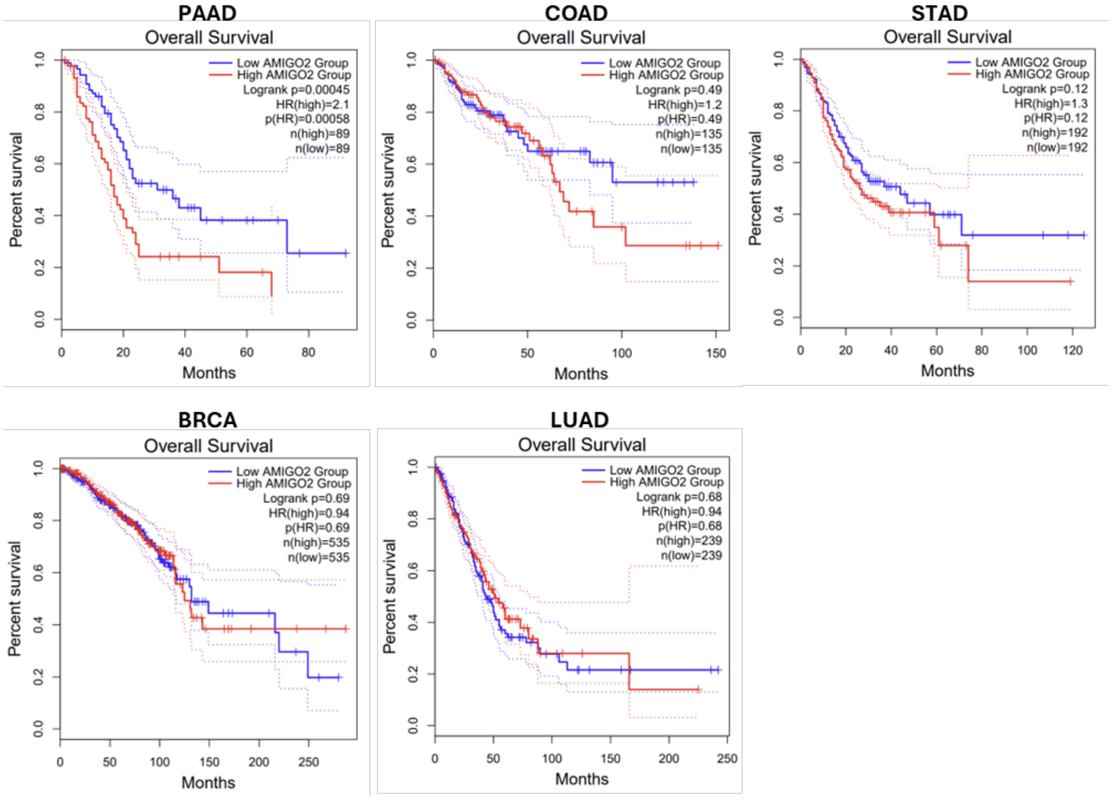

Supplementary Figure S1.

Pan-cancer survival analysis of AMIGO2 expression across TCGA cohorts. Kaplan–Meier curves show overall survival stratified by high vs. low AMIGO2 expression in pancreatic adenocarcinoma (PAAD), colon adenocarcinoma (COAD), stomach adenocarcinoma (STAD), breast cancer (BRCA), and lung adenocarcinoma (LUAD). Only PAAD exhibited a significant survival association (HR = 2.21, p = 4.5 × 10^{-4}).

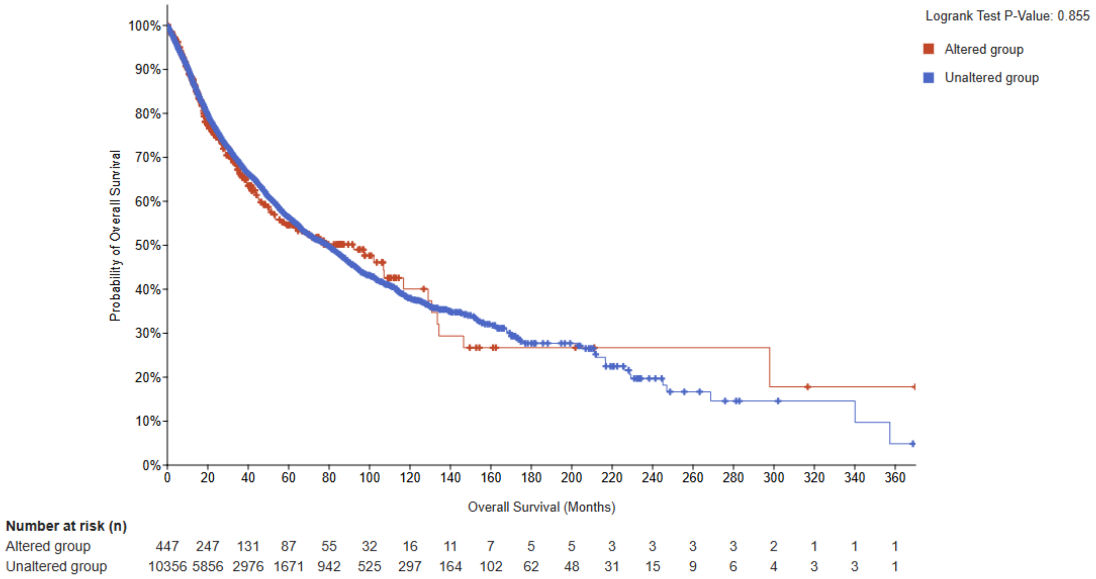

Supplementary Figure S2.

Combined alteration analysis of the AMIGO family (AMIGO1, AMIGO2, AMIGO3)

in the TCGA-PAAD cohort using cBioPortal. The survival curve shows no significant difference in overall survival between the altered and unaltered groups (Log-rank p = 0.855), indicating that AMIGO2 is the main prognostic driver among AMIGO family members in pancreatic adenocarcinoma.

Cancer type	n (total)	n (high)	n (low)	Hazard Ratio (HR, high vs. low)	Log-rank p-value	Prognostic impact
**PAAD**	178	89	89	**2.21**	**0.00045**	Significant, poorer OS
COAD	270	135	135	1.20	0.49	Not significant
STAD	384	192	192	1.30	0.12	Not significant
BRCA	1,070	535	535	0.94	0.69	Not significant
LUAD	478	239	239	0.94	0.68	Not significant

Supplementary Table S1. The correlation between AMIGO2 expression and distinct cancer types.
